# Muscular endurance and muscle metabolic responses to 8 weeks of omega‐3 polyunsaturated fatty acids supplementation

**DOI:** 10.14814/phy2.14546

**Published:** 2020-08-18

**Authors:** Takuma Morishima, Yosuke Tsuchiya, Hisashi Ueda, Eisuke Ochi

**Affiliations:** ^1^ Sports Research Center Hosei University Tokyo Japan; ^2^ Laboratory of Health and Sports Sciences Meiji Gakuin University Kanagawa Japan; ^3^ Faculty of Health and Medical Science Teikyo Heisei University Chiba Japan; ^4^ Faculty of Bioscience and Applied Chemistry Hosei University Tokyo Japan; ^5^ Graduate School of Sports and Health Studies Hosei University Tokyo Japan

**Keywords:** endurance performance, fish oil, muscle fatigue, oxygen saturation, supplementation

## Abstract

**Background:**

It has been well known that exercise training improves muscular endurance; however, whether nutritional strategies can be used to enhance muscular endurance remains unclear. Herein, we tested the hypothesis that 8 weeks of eicosapentaenoic acid (EPA) and docosahexaenoic acid (DHA) supplementation, known to promote oxygen availability and lipid metabolism, would attenuate muscular fatigue caused by numerous muscle contractions.

**Methods:**

Nineteen healthy men were randomly assigned to a placebo group (*n* = 9) and fish oil group (*n* = 10) in a double‐blind fashion. The fish oil group consumed EPA‐rich fish oil that contains 600‐mg EPA and 260‐mg DHA per day for 8 weeks. The placebo group received matching capsules for the same duration of time. After the 8‐week intervention, subjects performed muscular endurance test that was repeated knee extensions with weights equal to 40% of the subject's body weight.

**Results:**

Maximal repetitions to exhaustion were recorded. In addition, maximum isometric voluntary muscle contraction (MVC), muscle metabolism using near‐infrared spectroscopy, and blood lactate were measured during the test. Subjects in both groups reached exhaustion after the muscular endurance test, while the maximal repetitions did not differ between the groups. Similarly, there is no significant difference in oxygen saturation in muscle tissue (StO2), an index of muscle oxygen availability, between the groups. Also, MVC and blood lactate did not change between groups.

**Conclusion:**

In conclusion, the present study provided evidence that muscle fatigue caused by knee extensions cannot be attenuated by EPA and DHA supplementation in healthy subjects.

## Graphical abstract

Although it has been well known that exercise training improves muscular endurance, whether nutritional strategies can be used to enhance muscular endurance remains unclear. We tested the hypothesis that 8 weeks of eicosapentaenoic acid (EPA) and docosahexaenoic acid (DHA) supplementation, known to promote oxygen availability and lipid metabolism, would attenuate muscular fatigue caused by numerous muscle contractions. We confirmed that muscle fatigue caused by knee extensions cannot be attenuated by EPA and DHA supplementation in healthy subjects.

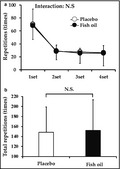

## 1 INTRODUCTION

Muscular endurance in response to repeated bouts of exercise is important to maintain exercise performance, particularly later on in various types of sports. Improved muscular endurance is associated with several physiological factors in skeletal muscle, such as increase in glycogen content, oxygen availability, buffer capacity, and metabolic shift toward an increased lipid metabolism (Clavel, Farout, Briand, Briand, & Jouanel, 2002; di Prampero, Atchou, Bruckner, & Moia, 1986; Joyner & Coyle, 2008). While exercise training is well reported to improve muscular endurance (Garber et al., 2011), little is known about the effects of nutritional intervention on muscular endurance.

Fish oil contains omega‐3 polyunsaturated fatty acids, including eicosapentaenoic acid (EPA) and docosahexaenoic acid (DHA). Recently, there has been growing evidence to suggest that oral EPA and DHA supplementation enhance endurance capacity by promoting oxygen availability and lipid metabolism (Clark, Monahan, & Drew, 2016; Logan & Spriet, 2015). Indeed, the 6–8 weeks of EPA and DHA supplementation has been reported to increase oxygen uptake and fat oxidation during submaximal pedaling test (Kawabata et al., 2014). Improved endurance performance and lipid metabolism following EPA and DHA supplementation were also observed in rat skeletal muscle (Clavel et al., 2002). Clavel et al. (2002) also observed an increase in cytoplasmic fatty acid‐binding protein (FABPc) in skeletal muscle in the fish oil supplementation group compared with the swimming training alone group. Since increase in FABPc was associated with an increase of citrate synthase activity but not lactate dehydrogenase activity, the improvement of muscular endurance, which is the oxidative capacity of skeletal muscle induced by exercise, might be associated with the FABPc.

In addition, we recently demonstrated that 8‐week EPA and DHA supplementation could attenuate the reduction of muscle work output caused by repeated maximal concentric muscle contractions (Ochi, Yanagimoto, Morishima, & Tsuchiya, 2018). Our previous study firstly proposed that EPA and DHA supplementation could increase muscular endurance in human. However, participants performed five sets of six maximal concentric contractions; thus, the total number of muscle contractions was only 30. Therefore, it remains unknown whether EPA and DHA supplementation are effective in sustaining muscle work output when muscle contractions are repeated numerous times with low‐to‐moderate intensity.

In the present study, we examined whether 8‐week EPA and DHA supplementation could be beneficial in maintaining muscle work output in response to numerous muscle contractions. Participants were divided into two groups (placebo and fish oil groups), and all‐out resistance exercise using low‐to‐moderate intensity with high repetitions was performed after 8 weeks of either placebo or EPA and DHA interventions. We hypothesized that muscular endurance would be enhanced by EPA and DHA supplementation.

## 2 MATERIALS AND METHODS

### 2.1 Subjects

Nineteen healthy men participated in the present study (age: 20.8 ± 1.5 years, height: 172.0 ± 6.7 cm, body mass: 67.0 ± 10.4 kg, BMI: 22.6 ± 2.9). Subjects had not participated in any regular resistance training for at least 1 year prior to this study. Participants were instructed to avoid any interventions that influence the outcome measures shown below, such as massage, stretching, and use of nutritional and NSAIDs during the experimental period. In addition, participants did not engage in any exercise. All participants provided an informed consent to participate in the present study that had been approved by a local institutional review board. The present study was performed in conformity with the policy statement about the use of human participants by Declaration of Helsinki; it was approved by the Ethics Committee for Human Experiments at Hosei University (ID: S2017‐01); and has been registered at the University Hospital Medical Information Network Clinical Trials Registry (UMIN‐CTR, identifier: UMIN000033141).

### 2.2 Study design

We used a double‐blind, placebo‐controlled, parallel‐group trial in the present study. Participants were randomly divided into two groups using a table of random numbers to avoid intergroup differences in age, BMI, and dietary EPA and DHA consumption. Participants were surveyed about their nutritional status during the 8‐ week supplementing period using the food frequency questionnaire that is based on food groups (FFQg version 3.5, Kenpakusha, Tokyo, Japan). The duration of supplementation was 57 days (including exercise days). For the intervention period and does, in a study by our group, it has been indicated that the 8‐ week supplementation with 600 mg of EPA and 260 mg of DHA has a beneficial effect on muscle work output in humans (Ochi et al., 2018). Sequence allocation concealment and blinding to participants and representative were sustained throughout the experimental period. At the end of study, we checked the subjective by day‐to‐day records and pill count. To improve the reliability of the pill count, participants were provided an excess number of pills and instructed to return any remaining pills at the end of the study. In addition, we measured serum fatty acid levels, including EPA, DHA, arachidonic acid (AA), and dihomo‐gamma‐linolenic acid (DGLA), before and after ingestion of the capsules. The blood samples at before and after 8‐ week supplementation were obtained as consistently as possible within subject, in morning hours between 9:00 and 11:00 a.m. On the day of leg muscle‐fatiguing testing, maximum isometric voluntary muscle contraction (MVC) torque, blood pressure, and blood lactate were assessed before exercise. After baseline measurement, participants performed leg muscle‐fatiguing testing. Oxygen saturation in muscle tissue (StO2), ventilation, and oxygen uptake were measured during exercise. All measurements were repeated immediately after exercise.

### 2.3 Supplements

The fish oil group (*n* = 10) consumed 2,400‐mg fish oil softgel capsules (Nippon Suisan Kai‐sha Ltd., Tokyo, Japan) per day, containing 600‐mg EPA and 260‐mg DHA. Previous works have demonstrated that more than 400 mg/day EPA and 200 mg/day DHA is necessary to attenuate muscle damage after exercise (DiLorenzo, Drager, & Rankin, 2014; Houghton & Onambele, 2012; Jouris, McDaniel, & Weiss, 2011; Tartibian, Maleki, & Abbasi, 2009, 2011; Tsuchiya, Yanagimoto, Nakazato, Hayamizu, & Ochi, 2016). Due to possible adverse side effects, we limited the amount of EPA and DHA to 3,000 mg per day that was based on the natural medicine comprehensive database (Administration, 2000). From the evidences of previous works and safety, we decided to use 400–2,000 mg/day EPA and 200–1,000 mg/day DHA in the present study. The placebo group (*n* = 9) ingested eight 300‐mg corn oil softgel capsules (Nippon Suisan Kaisha Ltd., Tokyo, Japan) per day (2,400 mg, not containing EPA and DHA). Participants ingested the supplements with water 30 min after meals.

### 2.4 Serum fatty acids

Participants fasted for 8 hr before blood samples were obtained from the forearm. Blood samples were allowed to clot at room temperature (25°C) and were then centrifuged at 3,000 rpm for 10 min at 4°C. Serum was extracted and stored at −20°C until analyzed. Serum levels of DGLA, AA, EPA, and DHA were measured using gas chromatography (GC). The test–retest reliability of the DGLA, AA, EPA, and DHA measurements showed coefficient of variation (CV) values of 5.6%, 4.9%, 5.1%, and 6.6%, respectively. CVs of serum fatty acid levels were published from the manufacturer. Participants did not take supplements prior to blood collection after intervention.

### 2.5 Experimental procedure

**FIGURE 1:**
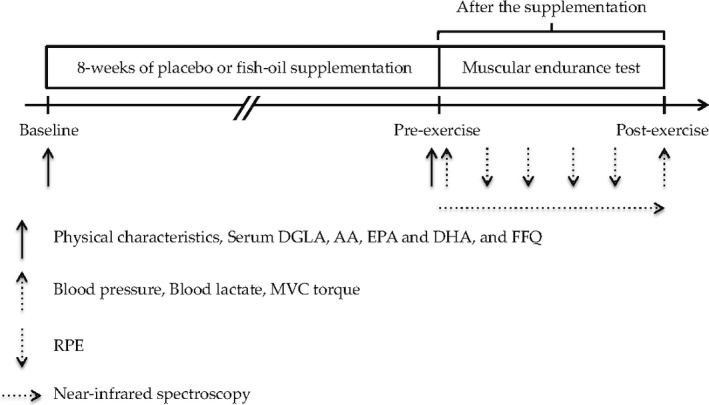
Experimental design

Figure 1 shows the experimental design. On the experimental day, participants were instructed to eat a light meal at least 2 hr prior to arriving at the laboratory. In addition, participants were asked to refrain from caffeine and alcohol for at least 10 hr as well as exercise for 24 hr prior to the study visit. All tests were performed in a temperature‐controlled room maintained at 23°C. Upon arrival, baseline MVC torque was assessed before the muscular endurance test. After the baseline measurements had been made, participants performed a muscular endurance test. During the test, StO2 and rating of perceived exertion (RPE) were measured. After the test, MVC torque measurement was repeated.

### 2.6 Muscular endurance test

The muscular endurance test consisted of a knee extensor load with weights equal to 40% of the participant's body weight. The protocol was based on a previous study by Sharma, Morris, and Adams (2015). Participants performed repeated bilateral knee extension exercise at 0.5‐Hz frequency (metronome assisted) for four sets in total. Each set was terminated when the participant failed to maintain adequate extensions at a frequency of 0.5 Hz. The resting periods among sets were 20, 30, and 40 s, respectively.

### 2.7 Measurements before, during, and after the muscular endurance test

#### 2.7.1 MVC torque

After a warm‐up exercise consisting of three to five knee extensions, MVC contraction torque of the nondominant (all participants’ nondominant leg was left) leg was measured using a custom‐made knee extension dynamometer, at a knee joint angle of 90° and hip joint angle of 70° flexion. Following sufficient familiarization and warm‐up, participants performed three 3‐s MVC trials with a 60 s rest between trials. We calculated MVC as the average knee extension torque over 0.25 s at the middle of contraction. The average MVC from the three trials was used for further analysis.

#### 2.7.2 Blood lactate

Blood lactate concentration was measured by a portable Lactate‐Pro analyzer (Arkray Inc., Kyoto, Japan) before and immediately after exercise, using fingertip blood samples (Chen, Nosaka, Lin, Chen, & Wu, 2009).

#### 2.7.3 Blood pressure

Participants were placed in a sitting position and brachial artery blood pressure was measured using a sphygmomanometer (HEM‐7130, OMRON, Kyoto, Japan) before and immediately after exercise. The test–retest reliability of the systolic and diastolic blood pressure measurements showed CV values of 6.1% and 8.1%, respectively.

#### 2.7.4 Near‐infrared spectroscopy (NIRS)

StO2 was recorded using an NIRS system (Hb 11; Astem Co., Kawasaki, Kanagawa, Japan) with two wavelengths (770 and 830 nm) and a probe consisting of one light source and two light receivers (Nagasawa, 2013). The present study measured the StO2 in vastus lateralis of the left leg. The distances between the source and the receivers were 2.0 and 3.0 cm, respectively, and the target site was muscular tissues at a depth of 1–2 cm from the skin surface (Nagasawa, 2013). The intramuscular muscle oxygenated hemoglobin/myoglobin (oxy‐Hb/Mb) and deoxygenated Hb/Mb (deoxy‐Hb/Mb) concentrations (millimoles) were estimated from the spatial slope of the light quantity detected by the two receivers using spatially resolved spectroscopy. Total‐Hb/Mb was defined as the total oxy‐Hb/Mb and deoxy‐Hb/Mb concentrations. StO2 was estimated using the oxy‐Hb/Mb to total‐Hb/Mb ratio. The oxy‐Hb/Mb, deoxy‐Hb/Mb, and total‐Hb/Mb concentrations assessed by NIRS have a considerable measurement error because of the effect of scatter factors caused by adipose thickness and muscle tissue. Since adipose tissue affects NIRS intensity (McCully & Hamaoka, 2000), the adipose thickness at the measurement points was determined using a skinfold caliper (EIYOKEN‐TYPE, PAT 376843, Meikosha, Tokyo, Japan) and was used to correct equations for the estimation of StO2. The validity of the StO2 value using these equations was verified previously (Nagasawa, 2013). The StO2 data were sampled at 2 Hz.

#### 2.7.5 Rating of perceived exertion

RPE was measured using a psychophysical category scale (Noble, Borg, Jacobs, Ceci, & Kaiser, 1983), with the participant rating the strength of his perception from 6 (“no exertion at all”) to 20 (“extremely strong”) (Loenneke, Balapur, Thrower, Barnes, & Pujol, 2011).

### 2.8 Statistical analyses

Changes in the dependent variables (repetitions during muscular endurance test, MVC torque, blood pressure, StO2, and blood samples) over time were compared between fish oil and placebo by a two‐way repeated measure analysis of variance (ANOVA). When a significant interaction or time effect was detected, we performed Bonferroni multiple comparison as a post hoc test. The CV was calculated by “standard deviations (*SD*)/means.” A significance level was set at *p* < .05. All variables were expressed as means ± standard deviations (*SD*).

## 3 RESULTS

Before the intervention, there were no differences in age or body composition between the fish oil group (age, 20.4 ± 0.7 years; height, 171.9 ± 7.5 cm; body mass, 68.5 ± 10.3 kg; BMI, 23.1 ± 2.7) and placebo group (age, 21.2 ± 2.0 years; height, 172.1 ± 6.1 cm; body mass, 65.3 ± 10.9 kg; BMI: 22.0 ± 3.0). The food frequency questionnaire revealed no significant differences in overall energy intake and consumption of protein, fat, carbohydrate, and omega‐3 fatty acids between the groups during the intervention period. These parameters did not change significantly during the intervention. Furthermore, as shown in Table 1, no significant changes were observed in the placebo group before and after the 8‐week supplementation in terms of DGLA, AA, EPA, and DHA levels. In the fish oil group, the EPA level increased after 8 weeks (*p* < .05). In addition, the DHA level increased after 8 weeks (*p* < .05). However, no significant difference was observed in the DGLA and AA levels. For comparison between groups, EPA and DHA levels were significantly higher in the fish oil group than in the placebo group after the 8‐week supplementation (*p* < .05).

**TABLE 1:** Physical characteristics of subjects and changes of serum dihomo‐gamma‐linolenic acid, arachidonic acid, eicosapentaenoic acid, and docosahexaenoic acid in fish oil and placebo groups at before and after 8‐week supplementation

		Before the 8‐week supplementation	After the 8‐week supplementation	ANOVA
Age(y)	Placebo		21.2 ± 2.0	
	Fish oil		20.4 ± 0.7	
Height (cm)	Placebo		172.1 ± 6.1	
	Fish oil		171.9 ± 7.5	
Weight (kg)	Placebo		65.3 ± 10.9	
	Fish oil		68.5 ± 10.3	
Body Mass Index	Placebo		22.0 ± 3.0	
	Fish oil		23.1 ± 2.7	
Dihomo‐gamma‐linolenic acid (μg/ml)	Placebo	35.0 ± 6.1	37.7 ± 6.9	Interaction: n.s.
	Fish oil	34.4 ± 9.6	31.9 ± 7.8	
Arachidonic acid (μg/ml)	Placebo	182.3 ± 33.7	186.8 ± 63.6	Interaction: n.s.
	Fish oil	180.4 ± 41.4	159.3 ± 23.1	
Eicosapentaenoic acid (μg/ml)	Placebo	20.9 ± 14.3	20.3 ± 10.1	Interaction: *p* < .05
	Fish oil	25.9 ± 19.9	60.2 ± 28.2^*^, ^#^	
Docosahexaenoic acid (μg/ml)	Placebo	64.8 ± 15.3	61.4 ± 14.4	Interaction: *p* < .05
	Fish oil	70.1 ± 23.9	82.6 ± 25.2^*^, ^#^	

Note

Mean ± *SD*.

* *p* < .05 versus pre.

# *p* < .05 versus Placebo group.

**FIGURE 2:**
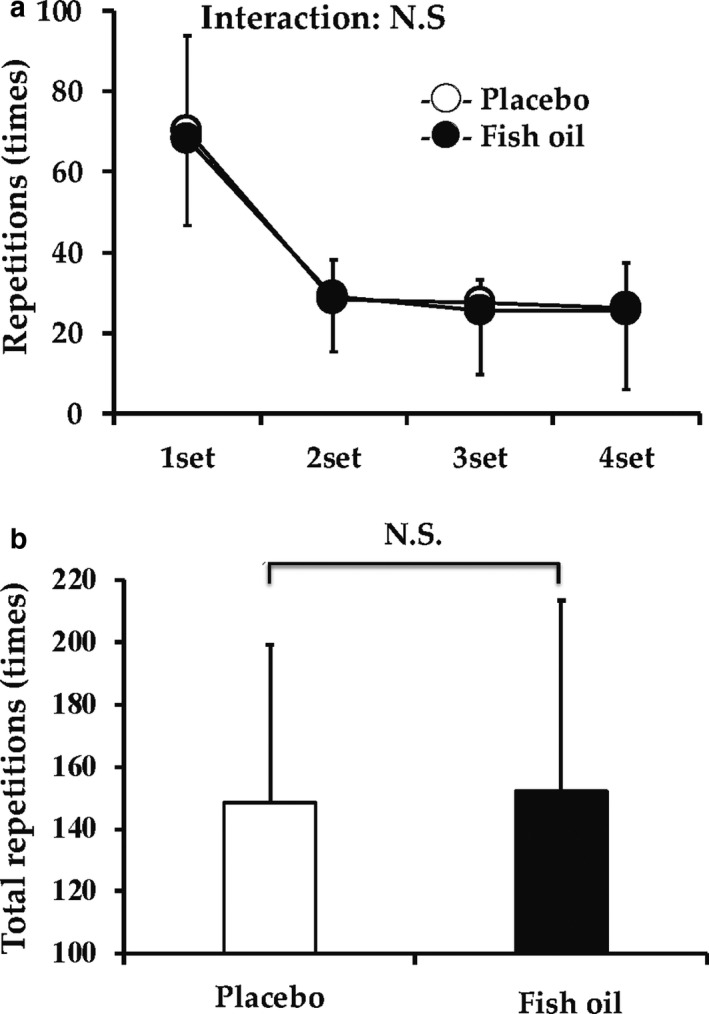
Repetitions to exhaustion for each set (a) and total (b) during muscular endurance test in fish oil and placebo groups. Data are expressed as mean ± *SD*

During the muscular endurance test, the maximal repetitions of first set in the placebo and fish oil groups were 70.3 ± 23.6 and 68.2 ± 25.7 times, respectively. The maximal repetitions were significantly reduced in the second, third, and fourth sets relative to first set in either group (*p* < .05). No significant differences were observed in the maximal repetitions in each set between groups (*p* > .05, Figure 2a). In addition, there were no significant differences in total (four sets) repetitions between the groups (Figure 2b).

As shown in Table 2, no significant differences were observed in RPEs for each set between the fish oil and placebo groups.

**TABLE 2:** Rating of perceived exertion (RPE) during the muscular endurance test in two groups

	Rating of perceived exertion (RPE)				
	1 set	2 sets	3 sets	4 sets	ANOVA
Placebo	15.8 ± 1.1	17.6 ± 1.3	18.8 ± 0.9	19.0 ± 1.4	Interaction: n.s.
Fish oil	16.9 ± 1.8	18.9 ± 0.6	19.5 ± 0.8	19.1 ± 1.7	Interaction: n.s.

Note

n.s. not significant.

**FIGURE 3:**
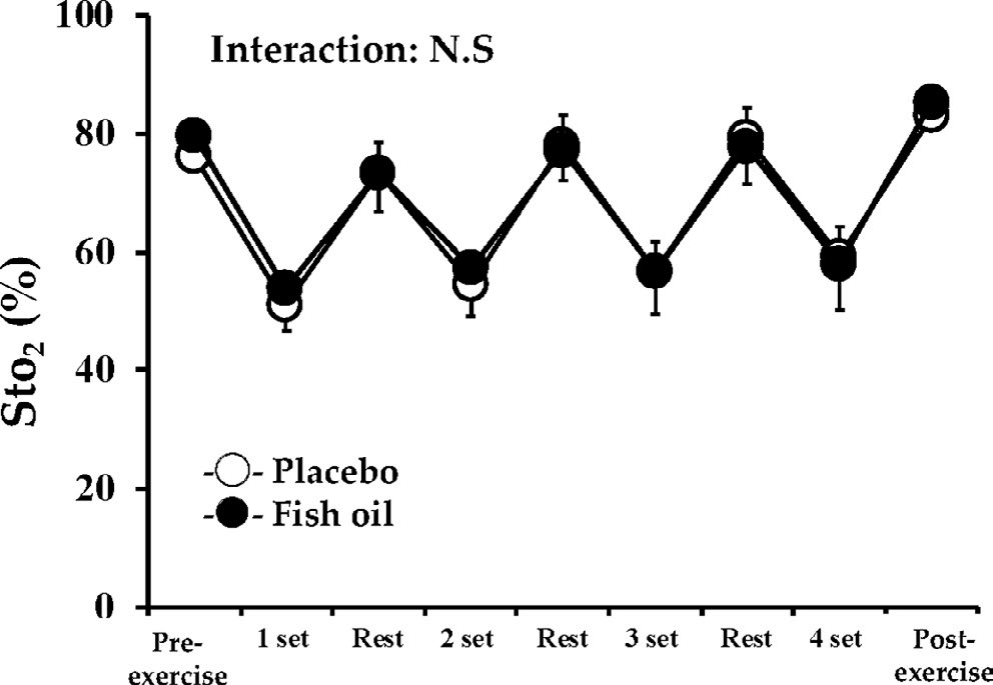
Oxygen saturation in muscle tissue (StO2) during muscular endurance test in fish oil and placebo groups. Data are expressed as mean ± *SD*

Oxygen saturation was markedly reduced during exercise in both groups (*p* < .05), and the reduction in oxygen saturation was restored during the resting period between sets. However, there was no significant difference in the time course of changes in StO2 between the fish oil and placebo groups (Figure 3).

**FIGURE 4:**
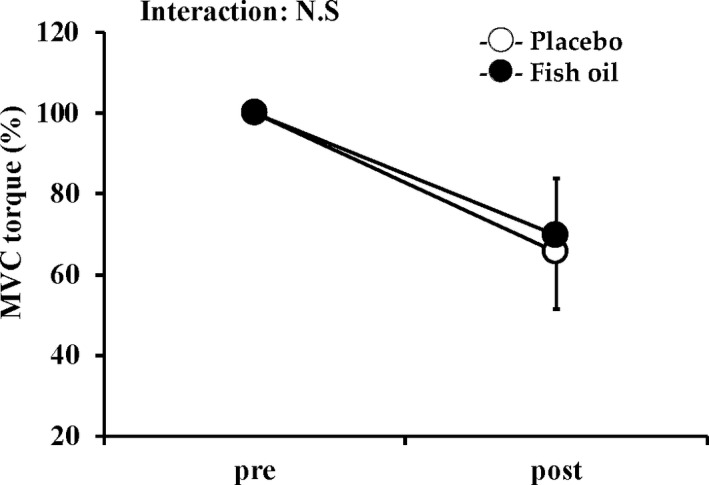
Changes in maximal voluntary isometric contraction (MVC) torque in fish oil and placebo groups before (pre‐exercise) and immediately after exercise (postexercise). Data are expressed as mean ± *SD*. n.s.; not significant

Prior to the muscular endurance test, there was no significant difference in MVC torques between the two groups (Figure 4, *p* > .05). As expected, MVC torques were significantly decreased after the test in both the fish oil (decreased by 32.6%) and placebo groups (decreased by 34.3%, *p* < .05) compared with baseline. The magnitude of the reduction of MVC torque did not differ between the groups (Figure 4).

Blood lactate increased (*p* < .05) immediately after exercise in both groups, but was not significantly different between the fish oil and placebo groups (Table 3).

**TABLE 3:** Blood pressure, and blood lactate at pre‐exercise and postexercise

		Pre‐exercise	Postexercise	ANOVA
Systolic blood pressure (mmHg)	Placebo	119.7 ± 10.7	153.8 ± 28.1^*^	Interaction: n.s.
	Fish oil	119.1 ± 9.1	151.6 ± 16.0^*^	Interaction: n.s.
Diastolic blood pressure (mmHg)	Placebo	66.2 ± 6.9	85.5 ± 31.3	Interaction: n.s.
	Fish oil	78.8 ± 11.5	97.0 ± 33.9	Interaction: n.s.
Blood lactate (mmol/l)	Placebo	1.3 ± 0.2	9.2 ± 1.6^*^	Interaction: n.s.
	Fish oil	1.1 ± 0.1	8.8 ± 1.9^*^	Interaction: n.s.

Note

Mean ± *SD*. n.s. not significant.

* *p* < .05 versus pre.

Systolic blood pressure increased (*p* < .05) immediately after exercise from the baseline in both groups, but was not significantly different between the two groups. Diastolic blood pressure did not increase in the fish oil and placebo groups after exercise, and did not differ between the groups (Table 3).

## 4 DISCUSSION

The main finding of the present study was that DHA and EPA supplementation did not attenuate the reduction of muscle work output in response to numerous muscle contractions. Indeed, we found that maximal repetitions to exhaustion, increased lactate, and blood pressure were similar in both the fish oil and placebo groups. In addition, oxygen saturation during exercise did not differ between the groups, suggesting that 8‐week supplementation with EPA and DHA does not affect muscle energy metabolism during repeated bouts of exercise using low‐to‐moderate intensity with high repetitions.

It was reported that the improved muscular endurance during exercise likely occurs by increasing oxygen availability and lipid metabolism, at least in part (Hill et al., 2007; Kawabata et al., 2014; Peoples & McLennan, 2010; Walser & Stebbins, 2008). In the present study, we examined whether EPA and DHA supplementation were effective to maintain muscle work output when muscles were exposed to exhaustive fatigue brought about by numerous repeated bouts of muscle contractions. This hypothesis was based on previous evidence that indicated a beneficial impact of EPA and DHA supplementation on oxygen availability and lipid metabolism (Peoples & McLennan, 2010). However, contrary to our hypothesis, EPA and DHA supplementation were not sufficient to attenuate muscle fatigue in the present study. We previously found that the reduction of muscle work output caused by five sets of six maximal pure concentric contractions (30 times in total) could be attenuated by 8‐week EPA and DHA supplementation (Ochi et al., 2018). Although the factors responsible for this inconsistent result between studies remain unknown, differences in exercise tests are thought to be the main reason. Our previous study used 30 concentric contractions as an exercise test, whereas participants performed all‐out resistance exercise using low‐to‐moderate intensity with high repetitions (including both concentric and eccentric contractions) in the present study, with the total number of muscle contractions reaching over 100. It is reasonable to suggest that the differences in the number of muscle contractions and exercise intensity (percentage MVC) are critical contributors to the inconsistent results. Therefore, we conclude that EPA and DHA supplementation does not improve muscular endurance when muscle contraction is repeated numerous times. Although it is still unclear whether another nutritional strategy would be useful to improve muscular endurance, present study suggests that attenuating muscle fatigue caused by numerous muscle contractions does not ease using nutritional intervention. Thus, exercise training appears to be the most effective strategy for improving muscular endurance. For example, resistance exercise using low‐intensity with high repetitions was shown to improve muscular endurance (Morishima, Tsuchiya, Iemitsu, & Ochi, 2018).

We did not observe any changes in muscle energy metabolism in the present study. Walser et al. (Walser, Giordano, & Stebbins, 2006) showed that 6‐week EPA and DHA supplementation enhanced brachial artery blood flow in response to submaximal handgrip exercise, suggesting that EPA and DHA supplementation may promote oxygen delivery into the active muscle during exercise. Kawabata et al. (Kawabata et al., 2014) tested V̇O_2_ under submaximal pedaling exercise with the same lactic acid conditions and then reported that ingested EPA and DHA had less V̇O_2_ during exercise than the placebo group. Furthermore, a negative correlation was found in this study between V̇O_2_ and red blood cell increases. Thus, it has been suggested that to improve exercise economy, there should be a relationship between EPA and DHA intake and increased red blood cells. Moreover, it has also been demonstrated that in a group that ingested EPA and DHA, RPE during exercise was decreased. Based on our previous study, we hypothesized that EPA and DHA supplementation may increase oxygen availability during resistance exercise. However, inconsistent with our hypothesis, there was no difference in StO2 during exercise between the groups. Given that maximal repetitions during muscular endurance test were similar between the two groups, it is reasonable that we did not observe any difference in StO2 in the present study. It is important to note that only young healthy participants were included in this study. Therefore, it is possible that endurance athletes may have benefits from this nutritional intervention.

The present study has several limitations. First, we used 860 mg of EPA and DHA supplementation per day. This dose is similar to that used in our previous studies (Ochi et al., 2018; Tsuchiya et al., 2016) that demonstrated that muscle damage and fatigue could be attenuated after exercise. However, local muscular endurance may require more doses of EPA and DHA. Therefore, it is necessary to investigate high amount of EPA and DHA. On the other hand, it is an important finding that this amount could not attenuate muscle fatigue caused by low‐to‐moderate intensity exercise. Second, knee extensor load was unified with weights equal to 40% of the participant's body weight in this study. However, the variation in performance was very large. Thus, future studies are need to use different types of muscular endurance test.

## 5 CONCLUSION

In conclusion, we provide evidence that muscle fatigue caused by numerous times of muscle contraction cannot be attenuated by EPA and DHA supplementation in healthy subjects. On the basis of these data, people should perform exercise training instead of relying on fish oil supplements for improvement of muscular endurance.

## CONFLICT OF INTEREST

No conflict of interest, financial, or otherwise are declared by the authors.
